# Spontaneous Free Peritoneal Perforation of an Infected Pancreatic Fluid Collection Managed with Laparoscopic Drainage and Necrosectomy

**DOI:** 10.1155/2021/5532096

**Published:** 2021-03-13

**Authors:** W. G. P. Kanchana, A. D. Dharmapala, B. K. Dassanayake, W. M. A. S. B. Wasala, K. B. Galketiya

**Affiliations:** ^1^Department of Surgery, Teaching Hospital Peradeniya, Sri Lanka; ^2^Department of Anaesthesia and Critical Care, Teaching Hospital Peradeniya, Sri Lanka

## Abstract

**Introduction:**

Free peritoneal perforation of pancreatic fluid collections is extremely rare and only few case reports exist in the literature. Many of these patients undergo emergency exploratory laparotomy due to sepsis and haemodynamic instability requiring sepsis control. The use of laparoscopic techniques in this circumstance is limited by the haemodynamic stability of the patient and the technical challenges. But effective laparoscopic management is associated with less morbidity to the patient. *Case Presentation*. A 28-year-old patient presented with worsening generalized abdominal pain with increased inflammatory markers. She required persistent inotropic support despite adequate fluid resuscitation. She had transient acute renal impairment and acute respiratory distress, which improved with noninvasive support. CECT (contrast-enhanced computed tomography) showed an infected pancreatic fluid collection with peritoneal free fluid. Aspiration of pelvic collection showed purulent fluid. Based on these clinical and imaging findings, she was diagnosed with a free peritoneal perforation of an infected pancreatic fluid collection. She underwent a laparoscopic drainage and necrosectomy of the infected pancreatic collection and peritoneal washout. She had a gradual recovery. All inotropes were omitted on the second day following surgery. She was sent to the ward from the ICU (intensive care unit) on the 4^th^ postoperative day.

**Conclusion:**

The laparoscopic approach is a viable option in managing ruptured pancreatic fluid collections when patient and technical factors are supportive. It reduces surgical morbidity, thereby reducing the overall strain on physiological reserves. When opted for laparoscopic drainage, the procedure must be guided by imaging findings. Multidisciplinary participation is critical in the overall management.

## 1. Introduction

Pancreatic fluid collections (PFC) are formed following episodes of acute pancreatitis. PFC following interstitial oedematous pancreatitis lead to pancreatic pseudocyst formation while acute necrotic collections (ANC) lead to the formation of walled-off necrosis (WON) after 4 weeks [1, 2]. Treatment strategy for PFC remains conservative in the acute phase unless infective complications lead to systemic effects and organ failure requiring control of sepsis [3].

Treatment of infected PFC depends on the haemodynamic stability and organ functions of the patient. Patients with no systemic complications are managed conservatively with antibiotics that penetrate pancreatic necrosis while drainage is recommended in patients with systemic effects that fail to improve with conservative measures [4]. Appropriate timing of the decision to intervene is critical in these patients as late interventions in severely compromised patients lead to futile outcomes.

Minimal invasive approaches are preferred in these patients as the systemic inflammatory response of a surgical intervention may drag the already compromised systemic reserves to their limits. But in a patient with free peritoneal perforation of a PFC, options available for control of sepsis are limited. Most patients in this circumstance undergo exploratory laparotomy to control intraperitoneal sepsis, which is associated with high morbidity and mortality [5].

The laparoscopic approach is sensible in this scenario, which allows drainage of intraperitoneal sepsis as well as approach PFC while reducing the morbidity of the procedure. Here, we present a patient who presented with spontaneous free peritoneal rupture of an infected PFC, who successfully underwent laparoscopic drainage and necrosectomy.

## 2. Case Presentation

A 28-year-old recently married lady with 8 years history of type I diabetes mellitus had presented to a local hospital with a one-day history of sudden onset generalized abdominal pain which was persistent and worsening over time. On examination, she had low blood pressure and low urine output. Despite fluid resuscitation, she required inotropic support to maintain blood pressure. Her abdomen was mildly tender to palpation with no significant guarding. She had no previous history of abdominal pain. An initial ultrasound scan of the abdomen revealed multiple pancreatic calcifications with mild to moderate ascites. She had high CRP (C-reactive protein) levels (258 mg/L), but her amylase level was <30 IU/L. Despite resuscitation, she developed acute kidney injury. She became oxygen-dependent and was developing features of respiratory failure as well. The patient was transferred to our unit for intensive care and further surgical management.

**Figure 1:**
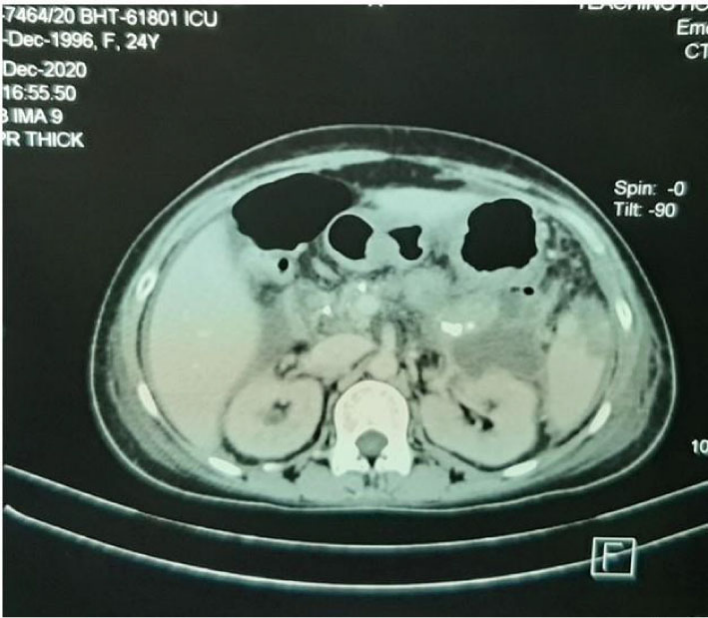
Pancreatic fluid collection at the tail of the pancreas with gas bubbles seen with in it suggestive of infected pancreatic fluid collection.

With continued resuscitation, her renal functions improved. But she was continued to be oxygen-dependent and required inotropic support. Contrast-enhanced computed tomography (CECT) of the abdomen revealed a pancreatic fluid collection (PFC) at the tail of the pancreas with gas formation within and severe peripancreatic fat standing and oedema, which was suggestive of an infected PFC in association with acute focal pancreatitis involving the pancreatic tail region (Figure 1). CECT also showed a moderate amount of free fluid in the abdomen with a pelvic fluid collection. Ultrasound-guided aspiration of pelvic collection revealed purulent fluid. Clinical picture and CECT findings were in favour of free peritoneal rupture of an infected PFC.

**Figure 2:**
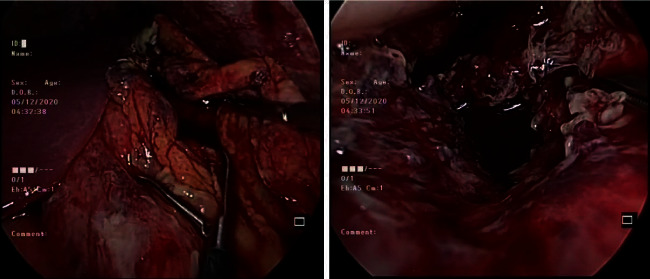
Widened opening at the gastro-colic omentum and the opening of the cavity of the necrotic collection following drainage.

**Figure 3:**
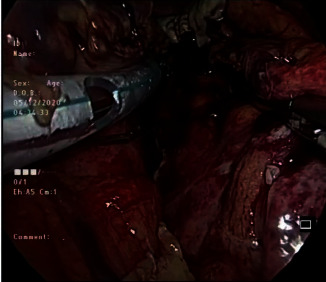
Drain placed into the cavity of the collection.

As she required control of sepsis within the peritoneal cavity as well as drainage of the infected PFC, laparoscopic drainage of PFC was planned. She was operated in a supine position. Pneumoperitoneum was achieved with the open Hassen technique. Laparoscopy revealed a moderate amount of purulent free peritoneal fluid with pus discharge from an opening in the gastrocolic omentum. Free peritoneal fluid and pelvic collection were drained. Opening in the gastrocolic omentum was widened to reveal the ruptured PFC (Figure 2). Cavity of the PFC was entered with blunt dissection, which revealed necrotic debris. Thorough lavage and drainage of the cavity was carried out. All dependent areas of the peritoneal cavity were inspected for residual collections. Following a thorough peritoneal lavage, wide bore tubes were placed into the cavity of the PFC and to the pelvis (Figure 3). A 32 Fr intercostal tube (IC) was placed into the cavity of the PFC, while a 14 Fr feeding tube was anchored inside it. This improvisation technique of the drainage tube allowed frequent flushing through the inner tube to prevent blockage of the larger drain tube. A single 32 Fr IC tube was placed into the pelvis.

Peritoneal fluid and necrotic tissue culture were positive for pure growth of coliform species. She had a gradual recovery. All inotropes were omitted on the second day following surgery. She was sent to the ward from the ICU on the 4^th^ postoperative day. Her inflammatory markers normalized. By one week from surgery, she was fully mobilized and was tolerating a normal diet. The drain that was placed into the pelvis was removed on the 4^th^ postoperative day as the drain amount was minimal.

Drain amylase levels from the drain placed into the cavity of the PFC was 15 U/L on the 3^rd^ postoperative day. Even though output from this drain was minimal since the 4^th^ postoperative day, it was kept until two weeks. She underwent a repeat CECT abdomen after 2 weeks from surgery, which showed resolution of the collection. At two months from surgery, she did not have any pain symptoms. As there was only a mild dilatation of the pancreatic duct and the patient did not have any pain symptoms, no further interventions were planned. She is scheduled for routine clinic visits for long-term follow-up.

## 3. Discussion

Pancreatic fluid collections are a frequent complication of acute pancreatitis [3]. Infection of these collections can occur in up to 40% of the cases, which can lead to systemic sepsis [6, 7]. These infected collections are initially managed conservatively unless the systemic effects are not responding to antibiotics and conservative measures [8]. When interventions for debridement and drainage of these acute collections are required, a step-up approach is preferred [9]. Percutaneous image-guided drains are initially placed followed by retroperitoneoscopic debridement. If all these minimal invasive methods fail, the last resort would be an open necrosectomy, which carries high morbidity and mortality rates [10, 11]. This step-up approach was developed over several decades from an era where open necrosectomy was the gold standard [9, 12, 13].

PFC may occur in chronic pancreatitis patients as well. One possible mechanism is following an acute episode of focal pancreatitis as seen in this patient. Another mechanism is pancreatic duct blowout due to main pancreatic duct obstruction [3, 5, 14]. Our patient had pancreatic calcifications involving head, neck, and body regions. But interestingly, she did not have the pain symptoms of chronic pancreatitis. But considering the long-standing history of type I diabetes, pancreatic calcifications and young age, the possibility of tropical calcific pancreatitis, need to be considered [15, 16].

Normal amylase levels in this patient can be explained by already depleted pancreatic parenchyma due to chronic pancreatic fibrosis and focal nature of the acute pancreatitis episode. Another possible explanation is a silent episode of acute pancreatitis, which lead to the formation of a pancreatic fluid collection, which later got infected producing the symptoms. With free peritoneal perforation, her symptoms worsened, and she went into severe sepsis.

Even though serum amylase levels were within normal limits, she was diagnosed as having acute chronic pancreatitis based on imaging evidence. CECT showed severe fat standing and oedema involving the pancreatic tail region. Fluid collection seen at the tail had a thin wall, which was suggestive of an acute fluid collection. As retroperitoneal air was seen within the collection, it amounted to CT grade E with a CT grade score of 4 in the CT severity index, which suggested a possible 35% morbidity and 6% mortality rate [1].

In contrast to a patient with infected pancreatic collection limited to the retroperitoneum, our patient had generalized peritonitis due to free peritoneal perforation of the collection. Thus, percutaneous interventions and retroperitoneoscopic debridement alone would not have controlled sepsis. Thus, the laparoscopic transperitoneal approach was considered suitable to deal with both the peritoneal contamination as well as the retroperitoneal collection.

Spontaneous intraperitoneal rupture of PFC is a rare presentation. Hence, only few case reports can be found in the literature. PubMed search revealed 5 cases of spontaneous free peritoneal perforation of PFC. Rocha et al. in 2016 presented two patients while Hui et al. presented one patient in 2019 [17, 18]. All these patients underwent exploratory laparotomy to control sepsis. Linn et al. very recently (2021) reported two cases where the laparoscopic approach was successfully used [19].

Laparoscopic drainage in these circumstances is rare due to a number of reasons including patient haemodynamic stability, concerns regarding adequacy of sepsis control through laparoscopic approach, and technical difficulties. Our patient was relatively stable at the time of surgery, and intraoperative findings were compatible with CECT findings. We could drain all the collections demonstrated in CECT, thus, the team was confident of adequate sepsis control. Ultimately, the patient had a successful outcome.

## 4. Conclusion

The laparoscopic approach is a viable option in managing ruptured pancreatic fluid collections when the clinical scenario and the technical factors are supportive. It reduces surgical morbidity, thereby reducing the overall strain on physiological reserves. The procedure needs to be planned, guided by the CECT images, while the overall management requires active multidisciplinary support.

## Data Availability

All patient data related to this case report is available on request.
